# Recent Developments in Halogen Free Flame Retardants for Epoxy Resins for Electrical and Electronic Applications

**DOI:** 10.3390/ma3084300

**Published:** 2010-08-11

**Authors:** Muriel Rakotomalala, Sebastian Wagner, Manfred Döring

**Affiliations:** Institute of Technical Chemistry, Karlsruhe Institute of Technology, Hermann-von-Helmholtz Platz 1, 76344 Eggenstein-Leopoldshafen, Germany; E-Mails: muriel.rakotomalala@kit.edu (M.R.); sebastian.wagner@kit.edu (S.W.)

**Keywords:** epoxy, fire retardant, phosphorus, printed wiring boards, composites

## Abstract

The recent implementation of new environmental legislations led to a change in the manufacturing of composites that has repercussions on printed wiring boards (PWB). This in turn led to alternate processing methods (e.g., lead-free soldering), which affected the required physical and chemical properties of the additives used to impart flame retardancy. This review will discuss the latest advancements in phosphorus containing flame retardants for electrical and electronic (EE) applications and compare them with commercially available ones. The mechanism of degradation and flame retardancy of phosphorus flame retardants in epoxy resins will also be discussed.

## 1. Introduction

In the 21^st^ century, polymer derived products are present everywhere in our daily life. Composite materials in particular have slowly replaced steel and aluminium alloys in a wide range of applications over the past decades [1,2]. Composite materials consist of an organic polymer matrix reinforced with either glass, carbon, aramide or natural fibres. In 2009, the estimated production of glass-fibre reinforced plastics in Europe was 815 kt [3]. Composite materials offer great physical, thermal, chemical and mechanical properties, while maintaining low density, high specific stiffness and strength, good fatigue endurance, corrosion resistance, good thermal insulation and low thermal expansion [4]. However, due to the organic nature of the polymer matrix and fibre (in the case of natural fibres), composite materials suffer from poor fire resistance.

**Figure 1:**
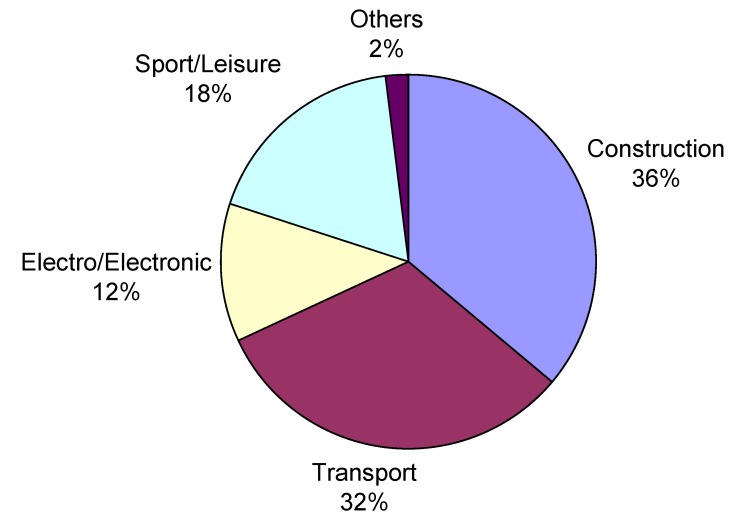
Application of glass-fibre reinforced composites in Europe (2009) [3].

**Scheme 1:**
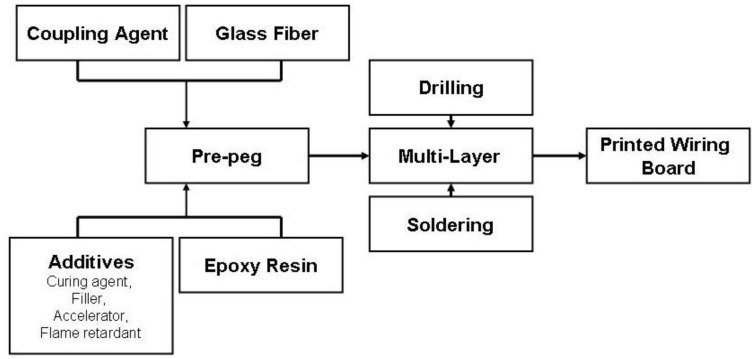
Assembly fabrication of FR-4 printed wiring boards.

The large variety of organic polymer matrices, both thermoset and thermoplastic, enables composite materials to be used for a wide range of applications, such as construction, transportation, aerospace, appliances and electrical and electronics (Figure 1) [5]. This review will focus on the epoxy polymers for electrical and electronic (EE) applications, which represents about 12% of the glass-fibre reinforced plastic market and also is one of the main applications of epoxy resin composites. In printed wiring boards (PWBs), 80% of composite materials are graded as FR-4 by the National Electrical Manufacturers Association (NEMA). FR-4 is a glass-fibre reinforced laminate of epoxies that meets defined flame retardancy standards (*i.e.*, UL 94-V0) [6,7,8]. During the first step of the fabrication of PWB, the glass is pre-treated with coupling agents such as organosilanes to improve adhesion between the inorganic glass and the organic resin [9]. In a separate container, the epoxy resin is mixed with additives such as curing agents, accelerators, fillers and flame retardants. The woven glass is then impregnated with the partially cured resin. The resulting partially cured reinforced material is known as pre-peg. Multiple pre-pegs are then thermally pressed to obtain a core. After assembly of several pre-pegs and cores, a layer of copper is electrodispersed on the surface to form a copper-clad laminate. Then holes are drilled and the laminate undergoes the soldering process (Scheme 1).

### 1.1. Epoxy Resins in Printed Wiring Boards

**Figure 2:**
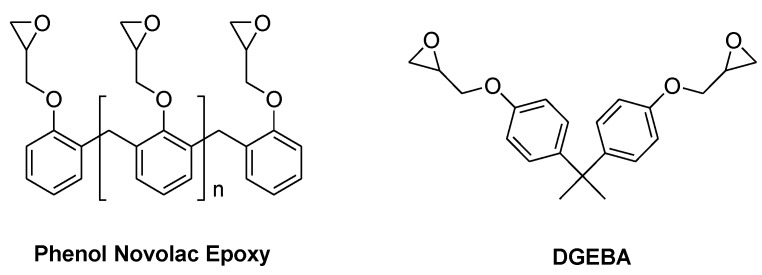
Commonly used epoxy resins for EE applications.

The principal epoxy resins used for EE applications are the bifunctional diglycidyl ester of bisphenol A (DGEBA) which is used for semiconductor encapsulation and the highly functional phenol novolac epoxy (PN-Epoxy) which is used for PWBs (Figure 2).

**Figure 3:**
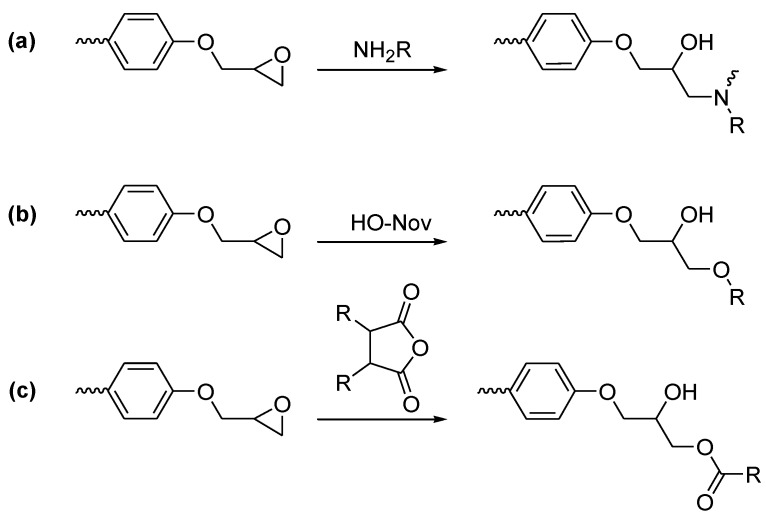
Newly formed bonds during curing of epoxy resins using: **(a)** amine, **(b)** phenol-novolac, or **(c)** anhydride as hardeners.

Epoxy resins in EE are commonly cured by reaction of the epoxy end group with amines **(a)**, phenol novolac **(b)** or anhydrides **(c)** (Figure 3). The polyaddition of the hardener with the epoxy resin will then generate hydroxyl groups that can contribute to increasing the cross-linking density of the cured resin [10].

**Figure 4:**
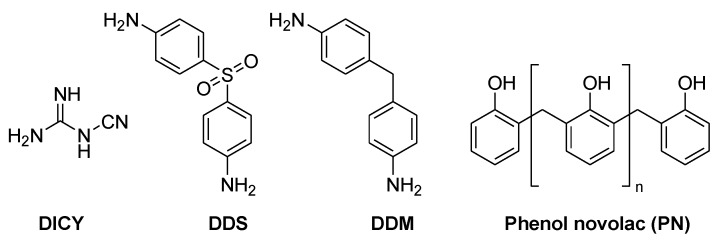
Regularly used hardeners for epoxy resins.

Different hardeners are used depending on the requirements of the application. In FR-4 laminates, dicyandiamide (DICY) and phenol novolac (PN) are mostly used (Figure 4) [5].

### 1.2. Thermal Degradation of Epoxy Resins

**Scheme 2:**
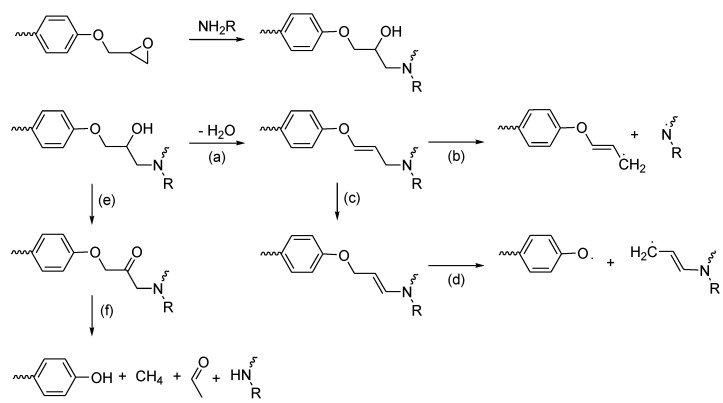
Thermal degradation paths of an amine cured epoxy resin.

Like other thermoset resins, when exposed to high temperatures (300–400 °C), the organic matrix of the cured epoxy laminate decomposes releasing heat, smoke, soot and toxic volatiles [11]. During the combustion of a polymer, there are generally four different reactions taking place in the condensed phase. The main reactions are end- or random-chain scissions that generate radical species. Simultaneously, various functional groups or atoms that are not part of the polymer backbone can be stripped off. Such a reaction is known as chain stripping. The last dominant reaction occurring is the cross-linking of the different radicals produced during the chain scission to form new thermally stable polymers or char. In the case of epoxy resins, the first step of thermal decomposition is the dehydration or dehydrogenation of the secondary alcohol formed during the cross-linking reaction to yield allylic amides (Scheme 2, **a**) [6,12,13,14]. The unsaturated moiety can then undergo isomerisation (**c**) followed by allylic-oxygen bond scission (**d**) [12]. In the case of amine hardeners the weak C-N bond formed during curing will then undergo allylic-nitrogen bond scission (**b**) to form volatile particles or contribute to charring [11].

Both the nature of the resin and the hardener determine the thermal stability of the cured resin. When a sample of DGEBA cured with DICY is exposed to fire, significant dripping is observed, which prevents it from passing UL 94-V0 rating. However, when novolac epoxy resins are cured using the same hardener, no dripping is observed. This can be justified by the difference in internal structure of the cured polymer. The high functionality of novolac epoxy resins leads to highly cross-linked structures whereas DGEBA leads to more linear and flexible polymer chains. Hence, under thermal stress, the flexible DGEBA polymer will undergo main chain scission resulting in the formation of smaller polymer chains. The shorter polymer chains formed have lower T_g_ and will therefore melt and drip off the burning polymer. In contrast, when the highly cross-linked cured novolac epoxy resin undergoes chain scission, the polymer chain length does not endure such a drastic shortening. Hence no dripping is observed.

### 1.3. The Use of Flame Retardants

**Scheme 3:**
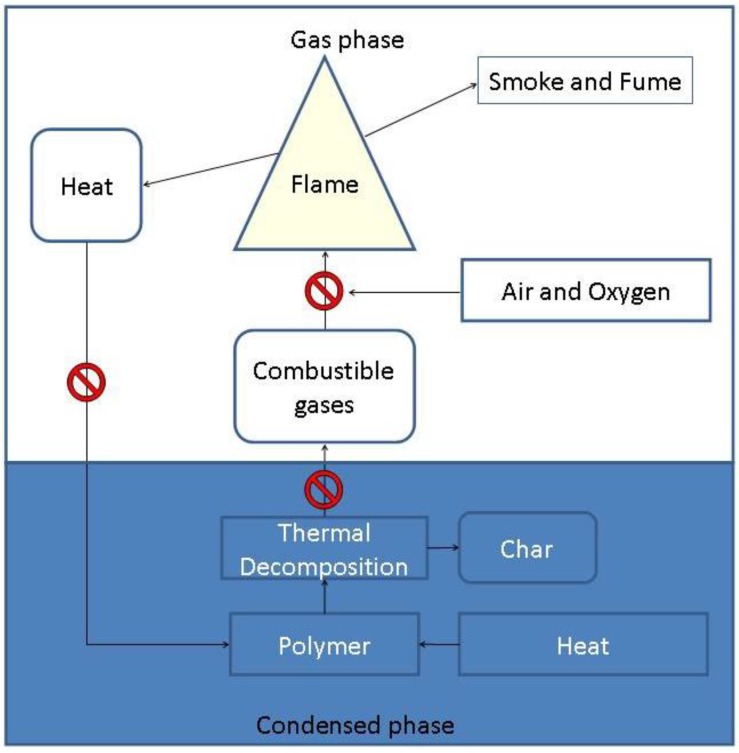
Combustion cycle of a polymer fire. Red marks represent the main approaches to extinguish a fire scenario.

The fire resistance of the cured resin can however be improved by the addition of a flame retardant. Thus, it is important to know that every application demands a different formulation (using different resins, hardeners and fire retardants). As shown in the flame cycle (Scheme 3) there are different steps where a fire scenario can be stopped.

**Scheme 4:**
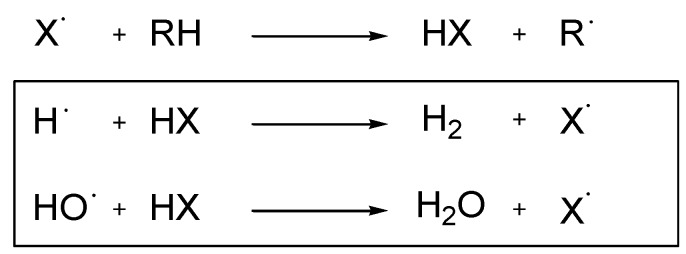
Gas phase reaction of halogenated flame retardants (X = Cl, Br).

A flame retardant can act in the gas phase by inhibition of the exothermic oxidation reaction in the flame via radical scavenging, thus reducing the energy feedback to the polymer surface. Scheme 4 demonstrates how halogenated flame retardants react via radical scavenging [15].

A flame retardant can also promote the formation of a thermal barrier (charring) at the surface of the condensed phase which blocks the release of gaseous fuel and prevents the transfer of heat back to the burning polymer [16]. An increased char yield results in a reduced amount of combustible gases reaching the flame which in turn leads to extinction. Flame retardants acting via the latter mechanism are known as condensed phase active because they catalyse the formation of char.

### 1.4. Challenges of Halogenated Flame Retardants

**Figure 5:**
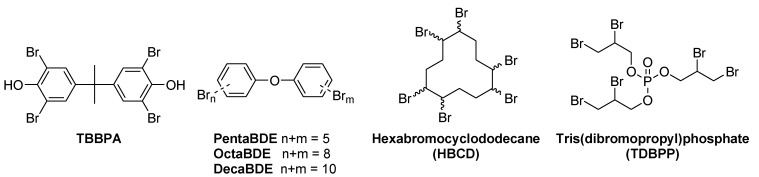
Halogenated flame retardants used for PWB.

Until recently, the majority of the flame retardants were halogen based and the most widely used one in epoxy resins for EE applications is the reactive tetrabromobisphenol A (TBBPA, Figure 5). When under thermal stress, bromine based flame retardants such as TBBPA are known to act as flame poisons by releasing volatile bromine radicals that scavenge hydrogen radicals in the flame to form non-flammable hydrogen bromide gas and in turn dilute the flammable oxidants (Scheme 4) [17]. This leads to an interruption in the flame cycle. Such flame retardants are known as gas phase active (Scheme 3).

It should be considered that the primary cause of death in household fires is smoke. The Underwriters Laboratories examined over 20 different combinations of materials found in common households. The result of the study showed that synthetic materials cause hotter fires and an increase in toxic smoke compared to natural furnishings [18]. It is a fact that consumer products, decorations and household objects in general, make home fires distinctively more dangerous. The average “escape time” for an occupant out of a burning home has dropped from 17 minutes in 1977 to 3 minutes in 2007. A fire retardant system has always limitations which, when exceeded, may lead to a sustained fire. The nature of the flame retardant added has a non-negligible impact on the toxicity of the fumes released [19]. Despite their benefits of slowing down the flame spread or reducing the fire growth, flame retardants can also increase the yield of toxic gases (such as carbon monoxide), or even decompose to toxic gases during a fire scenario (such as hydrogen bromide) [20]. Halogenated flame retardants in particular are often toxic or even carcinogenic by themselves. Environmental persistence and the ability to bio-accumulate add more concerns. By 2004 PentaBDE and OctaBDE (polybrominated diphenylether, Figure 5) were phased-out of production in North America and Europe because of the last mentioned doubts [21,22]. DecaBDE (Figure 5), on the other hand, remains one of the most widely used brominated flame retardants in consumer products until suitable replacements are found [23]. A recently released report of the Danish Ministry of the Environment summarises problems caused by DecaBDE and presents already available substitutes [24].

Since disputable additives can leach out of a polymer while being processed and/or while being used, there is always a potential health risk when such systems are used. In addition to the environmental and end-of-life issues, this led to strong efforts in replacing these halogenated systems. Some halogen compounds like tris(dibromopropyl) phosphate (TDBPP, Figure 5), used in fabrics, have been banned since the 1970’s and others will follow in the near future (e.g., hexabromocyclododecane, HBCD for polystyrene, Figure 5) [25]. Indeed, companies are well aware that with the continuing miniaturisation of electronics most devices are more likely to overheat. This in turn could cause self-ignition and increase the risk of fire. Beside miniaturisation there is also a trend towards mobile devices. In May 2005, laptop computers outsold regular desktop systems claiming 53% of all computer sales [26]. Laptops, PDAs and smart phones are particularly vulnerable to flammability because they carry their own power (and ignition) source.

**Scheme 5:**
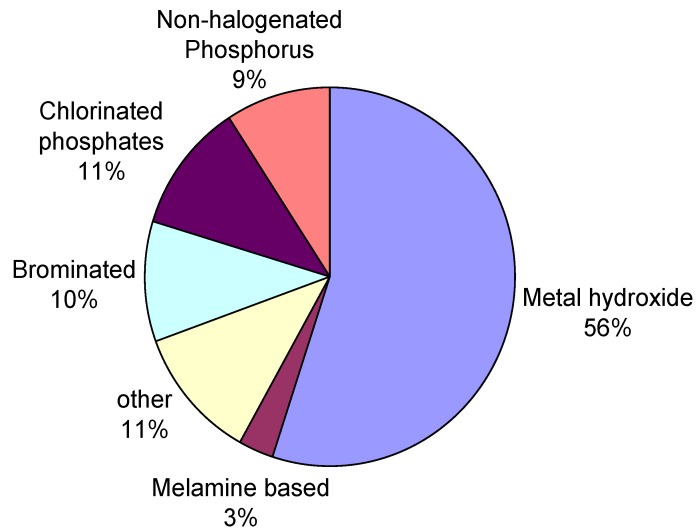
Market shares of different flame retardants for EE applications (2007) [5].

As seen in Scheme 5, halogenated flame retardants still represent a large portion of the flame retardants market for EE applications. However, as mentioned earlier, some brominated compounds will be gradually phased out to comply with the new environmental legislations (such as REACH, WEEE and RoHS). In February 2003, the EU introduced two new directives. The first directive, Waste Electrical and Electronic Equipment (WEEE), deals with recycling and further processing of electronic waste and stipulates separation of the bromine-containing plastics from the others [27]. The second guideline, Restriction of the use of certain Hazardous Substances (RoHS) restricts the use of different brominated compounds. Later in July 2006 heavy metals such as cadmium, chromium, mercury and lead were banned [28]. This affected the EE industry in particular because RoHS prohibits the use of lead in soldering. Alternatives to lead soldering require a raise in processing temperatures (~40 °C) which in turns demands a higher thermal stability from the additives used. Polybrominated biphenyl (PBB) and polybrominated diphenylether (BDE) were banned as flame retardants under EU´s RoHS and WEEE for the use in EE application. On the other hand, no risks were found when TBBPA was used. Nonetheless, under thermal stress unreacted TBBPA decomposes to bisphenol A which has been the subject of health concerns. Another important regulation is Registration, Evaluation and Authorization of Chemicals (REACH) [29]. According to REACH, only registered flame retardant chemicals with hazard data that underwent various tests concerning emissions and possible end-of-life issues can be used. An increasing number of companies, including Philips, Electrolux, Sony, Dell, Intel, Sharp, Apple and Hewlett Packard already started to introduce alternative flame retardants to their portfolio to meet these new regulations and to offer thoughtful products without health and environmental concerns [30]. Such flame retardants will be discussed in the following section.

## 2. Alternatives to Halogen Flame Retardants

The alternative compounds to halogen containing flame retardants for EE application will be discussed in this section. They can be separated into three groups:

- Inorganic flame retardants
- Nitrogen-based flame retardants
- Phosphorus-based flame retardants

### 2.1. Metal Hydroxides

Metal hydroxides such as, aluminium hydroxide (ATH) and magnesium hydroxide (MDH) have several positive effects when applied as a flame retardant. They are very cheap, easy to obtain, non toxic and environmentally friendly. Nonetheless very high loadings are required to obtain flame retardancy (~30–60%). Such high loadings have detrimental effects on the properties of the end product.

They have a strong tendency to react via a condensed phase mechanism. Metal hydroxides decompose to metal oxides and water, which is a highly endothermic reaction detracting energy from the ignition source (Equation 1: Endothermic reaction of ATH which leads to the release of water).

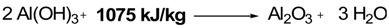
(1)

This flame retardancy mechanism is known as a heat sink. The released water evaporates, thus cooling the surface of the polymer and diluting the burnable gases at the same time [31]. The remaining metal oxides form a protective barrier on the polymer surface, shielding it against further decomposition (reducing the heat release rate) and reducing the amount of toxic gases released.

Aluminum-oxide-hydroxide (AlOOH or boehmite) has a much higher thermal stability and can be applied in epoxy systems that undergo lead-free soldering. As mentioned above, lead-free soldering required higher processing temperatures which demands higher thermal stability from the additives used. Prior to lead-free soldering a sample would pass the delamination test at 260 °C. However, the currently used system for PWB consisting out of a novolac epoxy resin and DICY as a hardener must endure 288 °C without delaminating. Metal hydroxides are often used as synergists with phosphorus based flame retardants (e.g., metal phosphinates).

### 2.2. Melamine Polyphosphate

**Figure 6:**
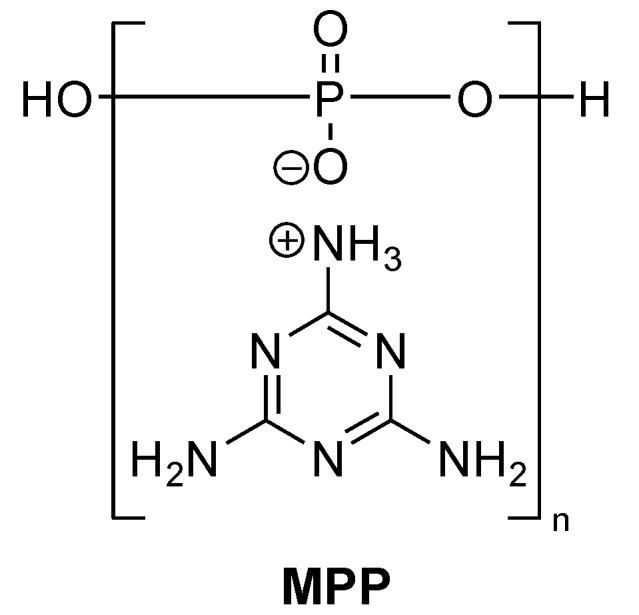
Melamine polyphosphate.

Melamine polyphosphate (MPP, Figure 6) is mostly used in combination with other flame retardants, such as metal phosphinates, metal hydroxides and phosphates [49]. It is characterised by its good thermal stability and a low impact on the glass transition temperature (T_g_). Under thermal stress, melamine derivatives decompose endothermically (heat sink) and release inert nitrogen gases (e.g., ammonia) that dilute oxygen and the flammable gases in the flame. Often phosphoric acid is also formed as a decomposition product and promotes the formation of insulating char on the surface of the polymer [32,33].

## 3. Phosphorus Flame Retardants

Red phosphorus, a long known and very effective fire retardant is mainly used in polyamides, polycarbonates and polyesters [34,35]. It is nontoxic and thermally stable up to 450 °C. Unlike white phosphorus, red phosphorus is not spontaneously flammable. As a polymer, red phosphorus breaks down during the process of fire to form P_2_ molecules that are active species in the gas phase. Red phosphorus reacts with moisture to form toxic phosphine gases, therefore it is important to provide suitable stabilisation and encapsulation. Since red phosphorus has an inherent colour, final products are limited to be brown and red. Toshiba described an adhesive formulation made from a blend of bisphenol A and cresol novolac epoxies using red phosphorus as a flame retardant [36]. They achieved UL 94-V0 rating with 4 wt % red phosphorus (encapsulated in a phenolic resin and coated with ATH) and 25 wt % ATH as a synergist.

**Figure 7:**
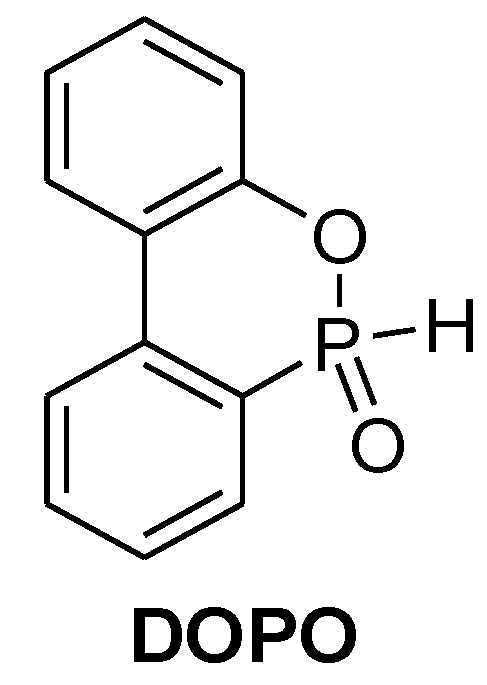
9,10-dihydro-9-oxa-10-phosphaphenanthrene-10-oxide.

The majority of literature on halogen free flame retardants focuses on phosphorus based products, which are predicted to be the largest growing share of the flame retardant market. Phosphorus flame retardants (organic and inorganic) are in general not harmful and do not tend to form toxic gases since phosphorus is mostly locked into the char [40]. Under thermal stress, the major part of phosphorus is oxidised to phosphorus pentoxide (P_2_O_5_) which then hydrolyses to polyphosphoric acid (H_x_P_y_O_z_). Polyphosphoric acid in particular plays an important role in creating carbonaceous char. Phosphorus flame retardants that react via the gas phase form phosphorus containing radicals and gases such as PO and PO_2_ derivatives respectively. The newly formed PO and PO_2_ derivatives can be rapidly oxidised to P_2_O_5_ which in turn forms polyphosphoric acid. It was found that non-halogenated phosphorus flame retardants have an environmentally friendly profile [37]. The environmental, health and end-of-life properties of 9,10-dihydro-9-oxa-10-phosphaphenanthrene-10-oxide (**DOPO**, Figure 7), which will be discussed in more detail later on, were investigated very closely [38].

In contrast to uncoated red phosphorus, no release of phosphine gases (PH_3_) were observed and it was also possible to partly recycle the phosphorus into fertilisers. Nonetheless, there are some reports on toxic and carcinogenic organophosphorus compounds which were used in the past (e.g., tris(dibromopropyl) phosphate) [39]. Organophosphorus compounds provide good physical properties and require less loading compared to regular fillers (e.g., ATH). However, a broad application is only gradually taking place since these are still more expensive than conventionally used flame retardants (e.g., ATH or TBBPA). Nonetheless, the production of phosphorus flame retardants on industrial scale will contribute to reduce their price.

### 3.1. Mechanism of Phosphorus Flame Retardants

**Scheme 6:**
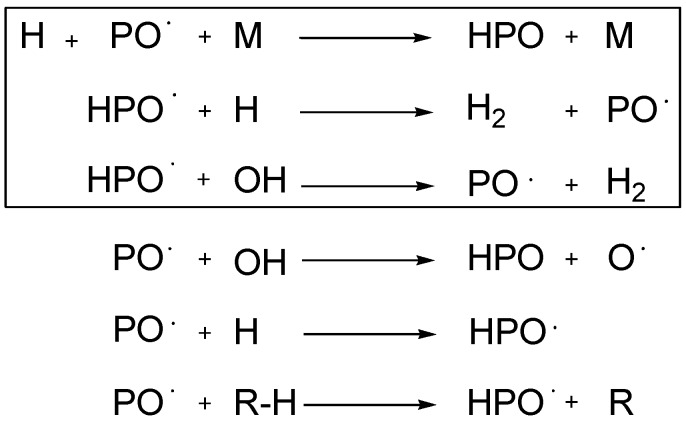
Elementary steps of the gas phase flame retardation by triphenylphosphine oxide and Exolit OP. The frame is highlighting the process of hydrogen scavenging. M is a third body species [40].

Although industrial and academic efforts led to numerous phosphorus-based flame retardant solutions, most of the results were based on empirical research. Considering that the degradation of an epoxy resin is strongly dependent on the nature of the resin and the hardener (and other additives) applied, there is no common mechanism of flame retardancy for phosphorus compounds. Phosphorus compounds that promote hydrogen recombination and scavenging of hydroxyl radicals by molecular phosphorus are also classified as gas phase active (Scheme 6) [40].

The release of phosphorus-containing volatiles that contribute to the extinction of the flame decreases with oxidation state of the phosphorus [41]. Another mode of gas phase activity is the dilution of the flammable gases by the release of inert/non-flammable gases (e.g., H_2_O for ATH).

Phosphorus based flame retardants tend to form polyphosphoric acids under thermal stress hence promoting the formation of thermally stable polymers (charing). A major benefit of phosphorus flame retardants is that both mechanisms are taking place [42,33]. It is possible to promote either mechanism through chemical tailoring.

There are two ways to render a polymer flame retardant. On the one hand the polymer can be blended with a flame retardant and on the other hand the flame retardant can be introduced to the polymer via a chemical reaction. The main difference between the two pathways it that the flame retardant is either blended as an additive (henceforth referred to as non-reactive FR) or covalently attached to the polymer (henceforth referred to as reactive FR).

### 3.2. Additive Flame Retardants

Both classes of flame retardants, the reactive and the non-reactive have various advantages in different applications. Additives represent the largest market share in flame retarded polymers (Scheme 5). To reach the desired effect, high loadings are necessary (up to 60 wt %), which often has a negative impact on the material and mechanical properties of the polymer [43]. The use of fillers has the great advantage that most additives (e.g., ATH) are very cheap and widely applicable.

**Figure 8:**
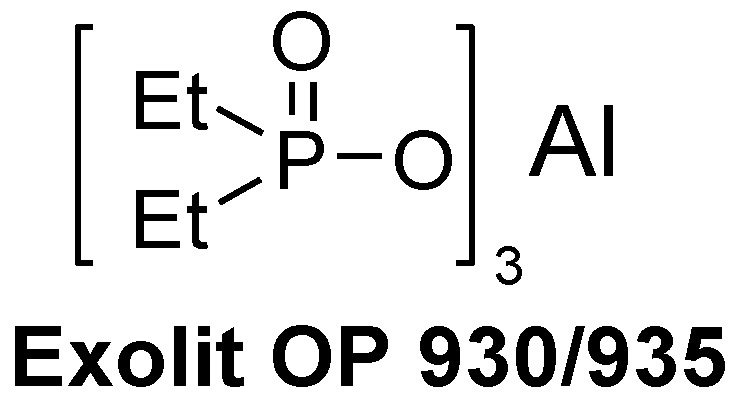
Commercially available aluminium phosphinates.

Metal salts of dialkyl phosphinates are known to be effective flame retardants since the late 1970s [44]. Clariant investigated a wide spectrum of zinc, aluminum and calcium salts of dialkyl phosphinates as flame retardants [45,46]. Aluminium diethyl phosphinates that were originally developed for glass-fibre reinforced polyamides and polyester achieved UL 94-V0 rating in with ~40 wt % additive [47]. Clariant initiated the production of aluminium salts of diethyl phosphinate, which are commercially available under the brand name Exolit OP 930 and Exolit OP 935 (Figure 8) [48]. They now find commercially promising application in PWBs [49]. The flame retardancy of Exolite OP 935 in the phenol novolac epoxy resin commercialised by Dow Chemicals (DEN 438) is summarised in Table 1.

**Table 1:** Required loading of Exolit OP 935 to achieve UL 94-V0 rating in DEN 438 cured with DICY/Fenuron. T_g_ measured by Differential Scanning Calorimetry (DSC) [8].

FR	FR-content (wt %)	Phosphorus-content (wt %)	UL 94-V rating	T_g_ (°C)
**Exolit OP 935**	9.5	2.0	V0	169
**Exolit OP 935 + MPP**	8 (4+4)	1.6	V0	179
**Exolit OP 935 + 30% Boehmite**	6.1	1.4	V0	171

Some of the key aspects of metal phosphinates are their high phosphorus content (~17%), good thermal stability (up to 320 °C) and lower affinity to moisture. Hydrolytic stability is especially important, since the release of phosphoric acids is not tolerated during extrusion or lead-free soldering because of acidic degradation. Schartel *et al.* investigated aluminium diethyl phosphinates (w/o melamine cyanurate as a synergist) as a flame retardant for polyesters (w/o glass-fibre). The results indicate that diethyl phosphinic acid is released in the gas phase during the decomposition of the polymer. UL 94-V0 rating could be achieved with a combined flame retardant loading of 20 wt % [50]. It has been reported that metal phosphinates are most effective in combination with a nitrogen synergist, such as melamine polyphosphate (MPP) [51].

**Figure 9:**
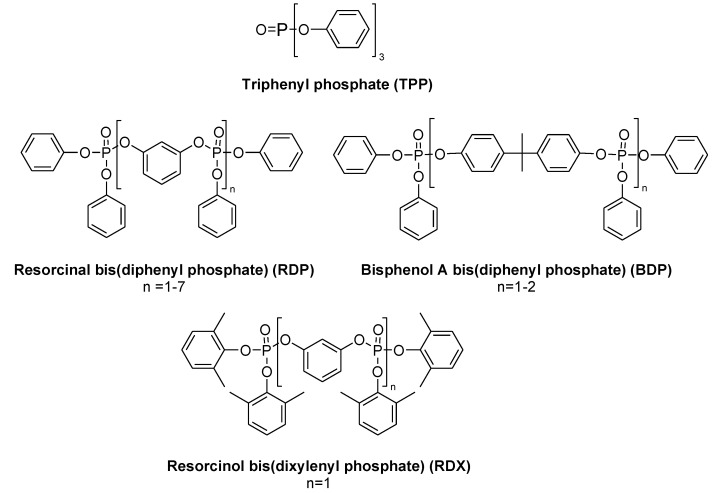
Commercially available phosphates.

Aromatic phosphates like triphenyl phosphate (TPP) are known to increase the flame retardancy of a polymer. A novolac epoxy resin hardened with DICY/Fenuron passes the UL 94-V0 test with only 1.6 wt % phosphorus (Table 3) [52]. A disadvantage of aromatic phosphates is that they are impaired by their reduced hydrolytic stability and often lead to a loss of clarity when blended into a polymer. Thus, they are mainly used as synergists in combination with bridged aromatic phosphates (e.g., RDP) [53]. Due to its spherical shape, TPP, which is a typical plasticiser, has a negative impact on the physical properties of the cured polymer. Compared to aromatic phosphates, bridged aromatic diphenyl phosphates have found a broad application beyond epoxy resins. The resorcinol and bisphenol A bridged diphenyl phosphates are available under the trade name Fyrolflex RDP and BDP respectively from ICL-IP. In Japan, Daihachi also commercialised RDX, a resorcinol bridged dixylyl phosphate under the trade name PX-200 (Figure 9).

Their main advantages are a good thermal stability (increasing from RDP to RDX), a high flame retardancy and low volatility. However, they are limited by potential plasticising effects and blooming (exudation), which can have a negative influence on electrical properties (current leak). Like TPP, RDP suffers from a hydrolytic instability but BDP and RDX are considerably more stable against moisture due the incorporation of bulkier groups compared to RDP.

On the other hand, large groups lead to a lesser phosphorus content. Therefore higher loadings are necessary. UL 94-V0 ratings can be achieved with 10–20 wt % additive, depending on the polymer and other synergists applied [52]. In the case of BDP UL 94-V0 rating could be achieved with 11.5 wt % flame retardant loading in novolac epoxy resin cured with DICY/Fenuron (Table 3). In contrast to TPP, bridged aromatic diphenyl phosphates are mostly active via a condensed phase mechanism. A strong char yield decreases fuel supply to the flame and reduces the heat release rate.

### 3.3. Reactive Phosphorus Flame Retardants

**Figure 10:**
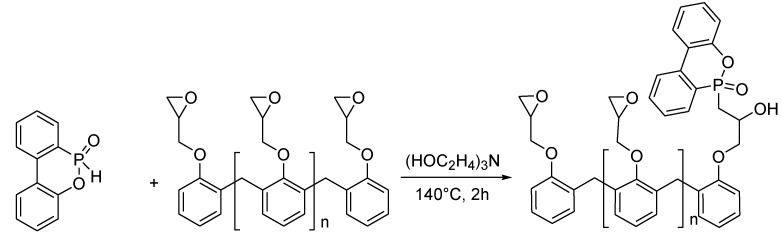
Rendering a novolac epoxy resin flame retardant via chemical incorporation of DOPO [54].

As mentioned above, the use of additive flame retardants, such as MPP, requires high loadings or the addition of a synergist in order to impart flame retardancy to epoxy resins used for EE applications. However, it should be specified that similar phosphorus contents are required for DOPO-based additives and for reactive DOPO derivatives to reach UL 94-V0 rating. Even though the reactive approach has yet to be adopted in industry, it has been subject to an ever growing interest from the academic community. Indeed, the reactive P-H bond of hydrogen phosphonates or phosphinates enables to covalently bind the flame retardant to the polymer chain by reaction with the epoxy functionality (Figure 10) [54].

This chemical modification renders the epoxy resin inherently flame retardant. Such an approach could theoretically allow lower phosphorus loadings than the additive approach and also eliminates the risk of flame retardant leaching from the polymer during the polymer processing. There has also been a growing interest toward phosphorus containing curing agents that would impart flame retardancy while acting as a cross-linker [55,56,57,58,59]. Such compounds will however not be discussed in this review. Until now, there has only been a limited amount of industrially relevant reactive phosphorus flame retardants.

9,10-Dihydro-9-oxa-10-phosphaphenanthrene 10-oxide (DOPO, Figure 7), which was developed by Sanko, was successfully pre-reacted with DGEBA (1–3% P) and cured with 4,4’-diamino- diphenylsulfone (DDS, Figure 4) and phenolic novolac (PN, Figure 4) [60,61]. UL 94-V0 rating was reached with both hardeners with 1.6 and 2.2% P-loading for DDS and PN respectively.

Only 1.6% P-loading was necessary in modified novolac epoxy resin cured with DICY/Fenuron to reach a UL 94-V0 rating [62]. It should be noted that DOPO is the first efficient halogen free flame retardant for novolac-based epoxy systems. It was recently reported that the addition of the inexpensive Boehmit (30 wt %) significantly reduced the required loading of DOPO (Table 2) [62].

**Table 2:** Synergistic effect of Boehmite and MPP in combination with DOPO in DEN 438 cured with DICY/Fenuron. T_g_ measured by Differential Scanning Calorimetry (DSC) [8,62].

FR	FR-content (wt %)	Phosphorus-content (wt %)	UL 94-V rating	T_g_ (°C)
**DOPO**	11.2	1.6	V0	155
**DOPO + MPP**	13.0 (6.5+6.5)	2.1	V0	157
**DOPO** **+ 30% Boehmite**	2.9	0.4	V0	168

**Scheme 7:**
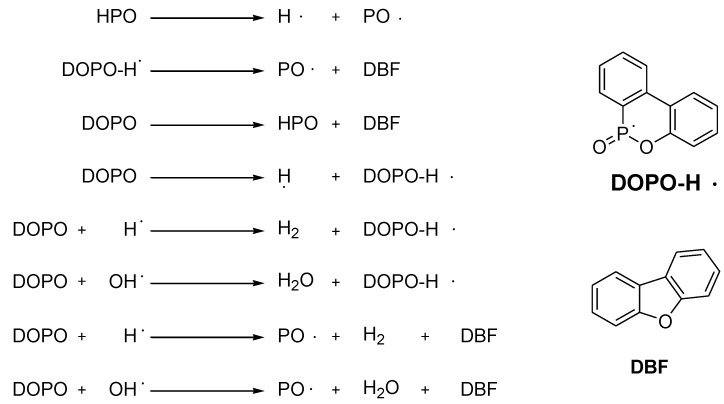
Proposed mechanism for flame retardant reactivity of **DOPO** in the gas phase.

DOPO is thought to act mainly through a gas phase mechanism. To confirm the gas phase activity of DOPO several TD-MS (thermal desorption mass spectroscopy) experiments were carried out and validated by DFT calculations. It indicated that PO and HPO are the gas active specimens (Scheme 7) [63].

The reactivity of this mono-functional phosphinates results in a decrease of the functionality of the epoxy resin [64]. Such reduction has been shown to have a detrimental impact on the T_g_ of the cured resin which represent a significant drawback for lead-free soldering that require a T_g_ over 170 °C.

**Figure 11:**
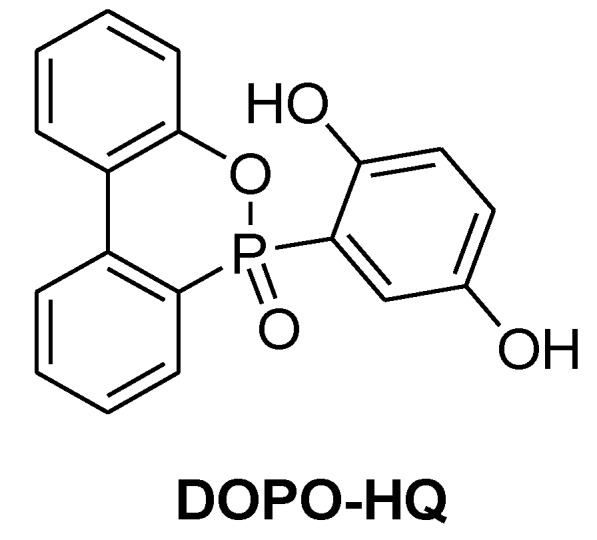
2-(6-Oxido-6H-dibenzo[c,e][1,2]oxa-phosphorin-6-yl)1,4-benzenediol.

In order to prevent the reduction of functionality of the epoxy resin, DOPO was reacted with benzoquinone to yield the bifunctional 2-(6-oxido-6*H*-dibenzo[c,e][1,2]oxaphosphorin-6-yl)1,4-benzenediol (DOPO-HQ, Figure 11) [65]. This hydroquinone product is currently commercialised by Sanko in Japan as HCA-HQ. When DOPO-HQ was pre-reacted with DGEBA only 2.1%P were required to impart flame retardancy. However, the thermal stability of the chain elongated DGEBA is similar to the unmodified cured resin, (T_d_ (5%, in air) = 397 °C unmodified vs. 401 °C FR). DOPO-HQ was also pre-reacted with a cresol novolac to yield a flame retarded resin with 1.1%P when cured with phenol novolac [66].

**Figure 12:**
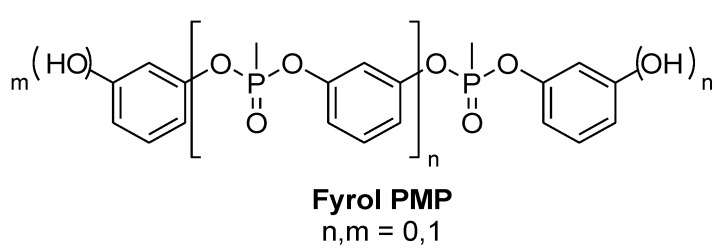
Fyrol PMP.

The terminal hydroxyl groups of poly(1,3-phenylene methylphosphonate) allow it to be reacted with epoxy resins and act as a flame retardant curing agent (Figure 12) [67]. Poly(1,3-phenylene methylphosphonate) is currently commercialised as a flame retardant for EE applications by ICL-IP as Fyrol PMP. Its thermal stability and high phosphorus content make it a good substitute for TBBPA [68]. When a novolac epoxy resin is cured with Fyrol PMP (20 wt %) in presence of ATH (35 wt %) a UL 94-V0 rating can be reached [69].

## 4. Recent Development in Phosphorus Flame Retardants

### 4.1. Non-reactive Phosphorus Flame Retardants

**Figure 13:**
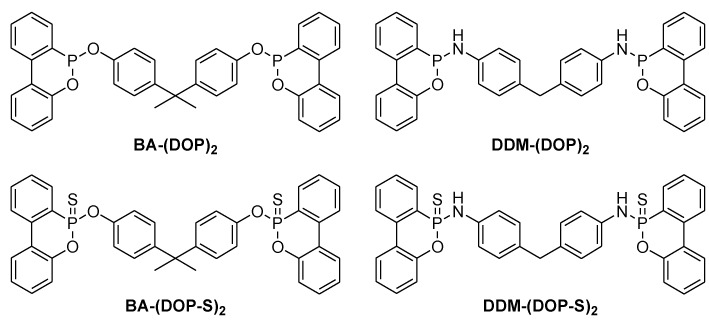
Bisphenol A and diaminodiphenylmethane bridged derivatives of DOPO.

The majority of recent results in academia and industry concerning phosphorus based flame retardants focuses on the reactive ones. However non-reactive flame retardants such as ATH still have an unparalleled cost-to-benefit ratio. On the other hand they often decrease the glass transition temperature which renders a formulation unsuitable for lead-free soldering. To avoid leaching, non-reactive phosphorus flame retardants should have a salt or polymer – like structure. Molecules like TPP have a small and compact geometry and are known to have a plasticising effect. The bisphenol A bridged diphenyl phosphate (BDP) has a much lower impact on the glass transition temperature which is a result of the rod-like geometry of the oligomere. This interesting fact was investigated more closely and it was found that bisphenol A and diaminodiphenylmethane (DDM) bridged derivatives of DOPO have a similar characteristic (Figure 13) [52].

The additives were blended into a novolac epoxy resin and hardened with DICY/Fenuron. The most promising results were found for DDM-(DOP)_2_, BA-(DOP)_2_, DDM-(DOP-S)_2_ and BA-(DOP-S)_2_ which all reached UL 94-V0 with 0.8, 1.0, 1.2 and 1.4 wt % phosphorus respectively.(Table 3)

**Figure 14:**
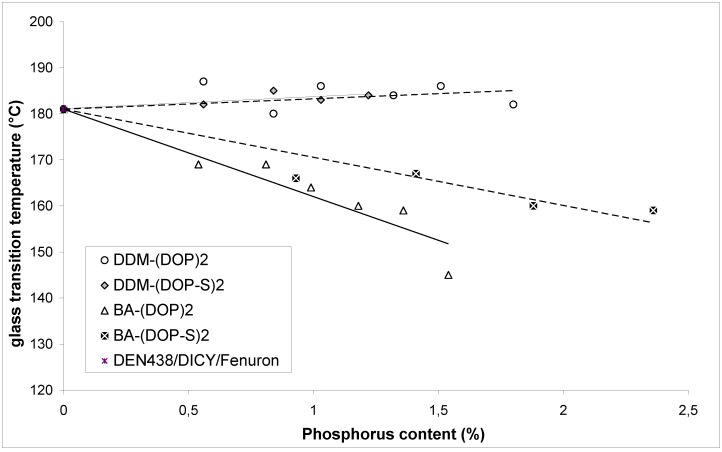
T_g_ *vs.* phosphorus content of DEN438 samples cured with DICY/Fenuron [52].

It was also found that trivalent phosphorus additives based on DOPO were inherently much better flame retardants than their oxidised pentavalent derivatives. Compared to TPP and BDP, no significant influence on the T_g_ (DEN 438/DICY/Fenuron: T_g_ = 181 °C) was found for DDM-(DOP)_2_ and DDM-(DOP-S)_2_. BA-(DOP)_2_ and BA-(DOP-S)_2_ showed a noteworthy plasticising effect (Figure 14). The phosphoramidates DDM-(DOP)_2_ and DDM-(DOP-S)_2_ are very effective fire retardants and the first examples of phosphorus based additives that show no influence on the T_g_ of the final product.

**Table 3:** T_g_ of the neat resin (DEN 438/DICY/Fenuron) = 181 °C. T_g_ measured by Differential Scanning Calorimetry (DSC) [8,52].

FR	FR-content (wt %)	Phosphorus-content (wt %)	UL 94-V rating	T_g_ (°C)
**TPP**	16.81	1.6	V0	136
**BDP**	11.55	2.07	V0	157
**BA-(DOP)_2_**	10.02	0.99	V0	164
**BA-(DOP-S)_2_**	15.28	1.41	V0	167
**DDM-(DOP)_2_**	9.85	0.84	V0	180
**DDM-(DOP-S)_2_**	12.94	1.22	V0	184

**Figure 15:**
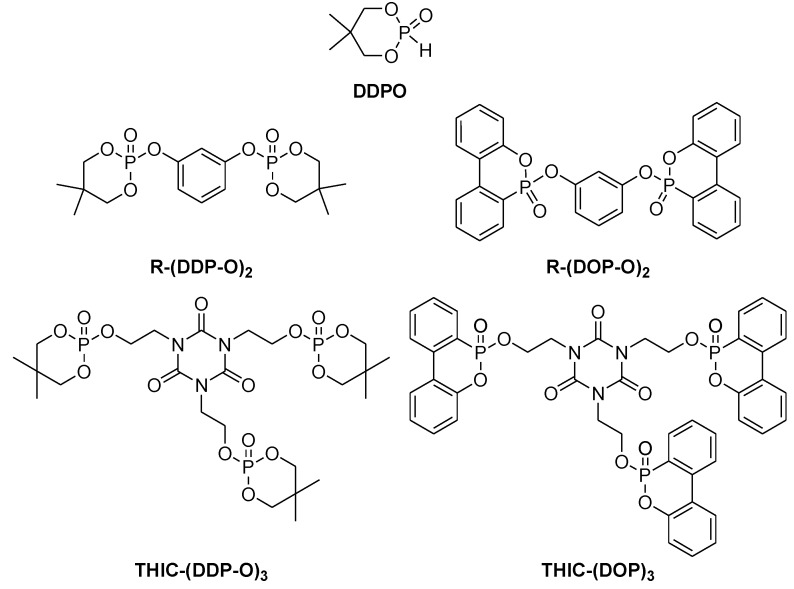
Tris(2-hydroxyethyl) isocyanurate and resorcinol bridged derivatives of DOPO and DDPO.

Beside the empirical search for new and effective flame retardants, it is also important to understand the structure property relationship of phosphorus and its chemical environment to rationally design and optimise flame retardants for specific applications. Recently, derivatives of DOPO and 5,5-Dimethyl-[1,3,2]dioxaphosphinane 2-oxide (DDPO) were synthesised and incorporated in two sets of resins, the novolac resin DEN 438 (hardened with DICY/Fenuron) and DGEBA (also hardened with DICY/Fenuron) (Figure 15) [70].

The results indicate that DOPO derivatives are preferable for epoxy resins with a high amount of aromatic subunits (such as DEN 438/DICY/Fenuron) and DDPO derivatives are most suitable for epoxy resins with a high amount of aliphatic hydrocarbons (such as DGEBA/DICY/Fenuron). In the case of DEN 438, DICY/Fenuron a gas phase mechanism is presumed to be dominating, therefore DOPO derivatives are the flame retardant of choice. For DGEBA/DICY/Fenuron, a condensed phase mechanism is proposed, making in R-(DDP-O)_2_ and THIC-(DDP-O)_3_ the most suited flame retardants. An increased char yield supports this mechanism. The resorcinol derivatives possess a rod-like geometry while the tris(2-hydroxyethyl) isocyanurate (THIC) bridged molecules adopt a star like geometry that both results in a negligible impact on the glass transition temperature. The size-effect of the flame retardant molecules was also discussed by Perez *et al*. [71,72] Table 4 summarises the results and UL 94 ratings.

**Table 4:** T_g_ of the neat resin (DEN 438/DICY/Fenuron) = 181 °C and (DGEBA/DICY/Fenuron) = 135 °C. T_g_ measured by Differential Scanning Calorimetry (DSC) [8,70].

	DEN 438 (DICY/Fenuron)		DGEBA (DICY)	
FR	Phosphorus-content for V0 (wt %)	T_g_ (°C)	Phosphorus-content for V0 (wt %)	T_g_ (°C)
**R-(DDP-O)_2_**	3.0 (n.c.)	-	2.0	135
**R-(DOP-O)_2_**	1.6	175	2.5 (n.c.)	-
**THIC-(DDP-O)_2_**	2.5	173	2.5	136
**THIC-(DOP-O)_2_**	1.5	176	2.5 (V2)	136

### 4.2. Reactive Phosphorus Flame Retardants

Since the discovery of the flame retardancy of DOPO in formulations used for EE applications, there has been an increasing number of reports about phosphacyclic derivatives. Even though DOPO can impart flame retardancy to novolac epoxy resin with low phosphorus content (~1.6%), the chemical incorporation of the flame retardant phosphacycle results in a lowering of the possible cross-linking. This results in a lower T_g_ which is detrimental for EE applications (e.g., soldering process). A common approach to solve the negative effect of chemically reducing the number of epoxy groups is to introduce bifunctional flame retardants that can act as chain elongators.

**Figure 16:**
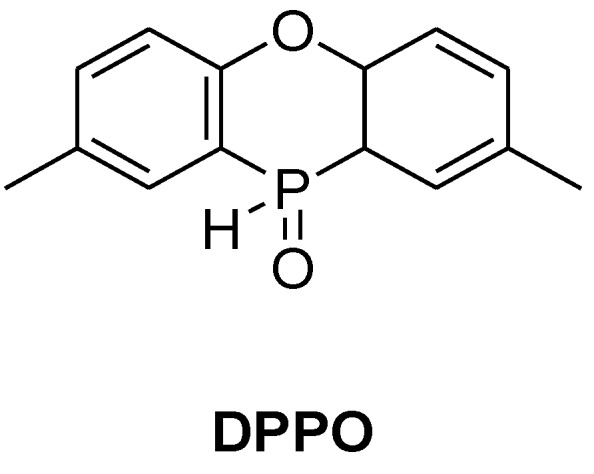
2,8-dimethyl-phenoxaphosphin-10-oxide.

Schäfer *et al.*, recently reported the synthesis and successful incorporation of 2,8-dimethyl-phenoxaphosphin-10-oxide (DPPO) in novolac epoxy resin (Figure 16) [73]. Similarly to DOPO, the T_g_ of the cured resin decreased with increasing phosphorus content however lower phosphorus content was required to impart UL 94-V0 rating (0.6%) when cured with DDM.

A large portion of the reports on reactive flame retardants focuses on DOPO-derivatives that could tackle the decrease of T_g_ resulting from chemical incorporation of the flame retardant [74]. The DOPO-HQ (Figure 11) has proven a promising replacement for DOPO as it can impart UL 94-V0 rating without severe impact on the T_g_ [75].

**Figure 17:**
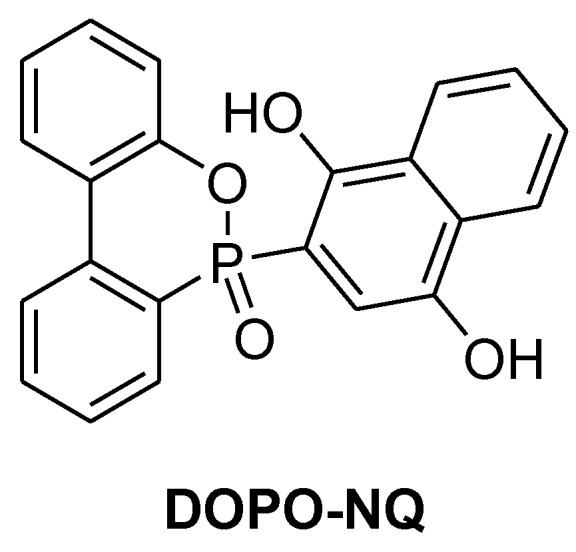
DOPO-NQ.

In a similar approach Ho *et al.* investigated the flame retardant properties of DOPO-NQ in DGEBA (Figure 17) [76]. The DOPO-NQ-containing epoxy resin cured with cyanate ester had higher T_g_ (162-207 °C) than the DOPO-HQ-containing one cured with DDS (164 °C) and UL 94-V0 rating were reached with ~2.1%P [77].

**Figure 18:**
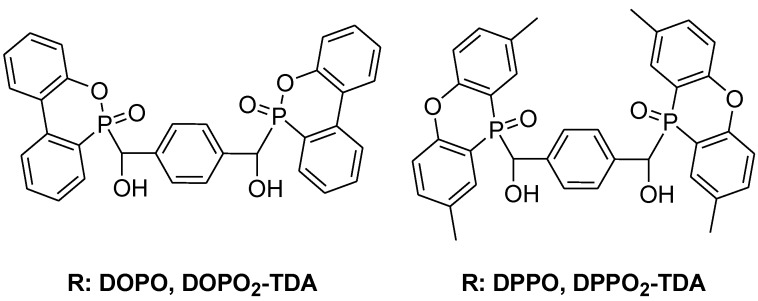
DOPO and DPPO adducts with terphathaldialdehyde.

The introduction of flame retardant molecules in the polymer backbone was also reported by Seibold *et al.* who reacted phosphorus-rich molecules DOPO and DPPO with terphathaldialdehyde to form new bifunctional flame retardants for PWB (Figure 18) [78]. The novolac epoxy resins cured with DDM and the bifunctional phosphorus molecules reached UL 94-V0 rating with 1.02 and 1.45% phosphorus for the DOPO and DPPO derivatives respectively. Both modified resins had an increased char yield when compared to the unmodified resin. (Table 5)

**Figure 19:**
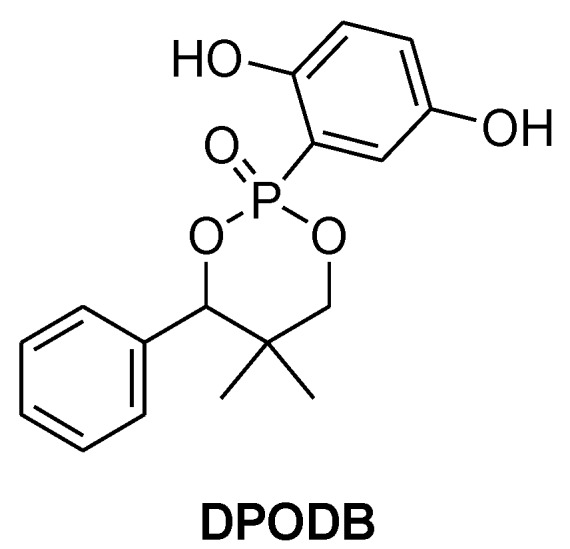
2-(5,5-dimethyl-4-phenyl-2-oxy-1,3,2-dioxaphosphorin-6-yl)-1,4-benzenediol.

Xia and *et al.* synthesised a phosphorus containing DGEBA-based monomer using 2-(5,5-dimethyl-4-phenyl-2-oxy-1,3,2-dioxaphosphorin-6-yl)-1,4-benzenediol (DPODB, Figure 19) as a chain elongator [79]. The newly synthesised monomer was then cured with cresol formaldehyde novolac resin and DDS. The cured resin reached UL 94-V0 rating and produced more char than the unmodified one (44% for FR resin *vs.* 27% for unmodified resin).

**Figure 20:**
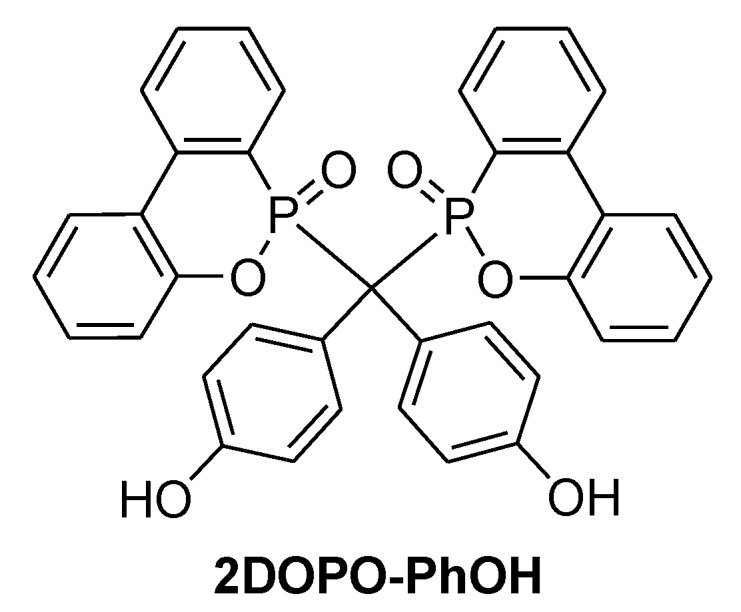
DOPO substituted chain elongator.

The reaction of a DOPO with dihydroxy benzophenone followed by addition of DGEBA yielded an elongated DGEBA resin (Figure 20). The elongated resin had a T_g_ of 131 °C and 148 °C when cured with DDM and DICY respectively [74].

**Figure 21:**
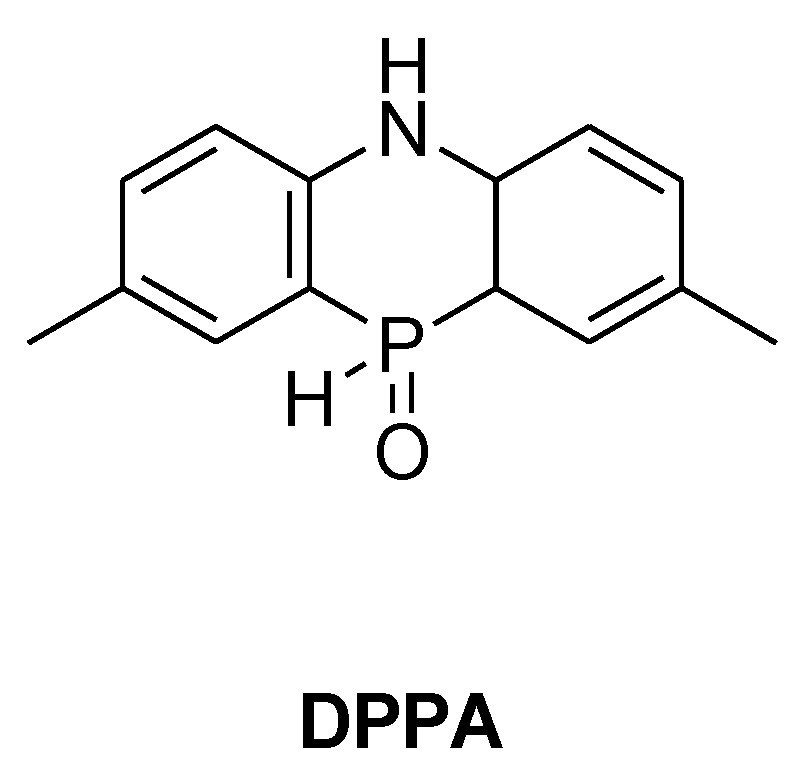
5,10-Dihydrophosphosazine-10-oxide.

**Figure 22:**
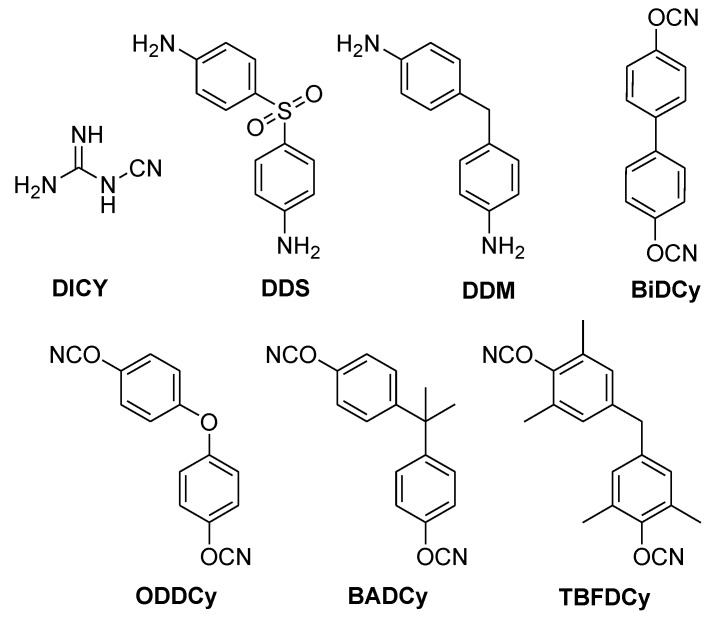
Hardeners used to cure epoxy resins (c*f.* Table 5).

In an effort to counter the effect of chemically adding the flame retardant on the T_g_, Schäfer *et al.* synthesised and reacted 5,10-dihydro-phosphosazine-10-oxide (DPPA, Figure 21) with DGEBA [80]. The resultant polymer was cured with DDM and reached UL 94-V0 rating with 3.2%P. The T_g_ of the DPPA-modified resin was lower than that of the unmodified DGEBA (131 °C *vs.* 184 °C) but was higher than the flame-retarded DOPO-modified resin. Thus the addition of the amine functionality counter balances the detrimental effect of reactively adding the phosphacycle to the epoxy resin. Table 5 summarises the results and Figure 22 presents the different hardeners used.

**Table 5:** Summary of reactive phosphorus flame retardant performance in epoxy resins. T_g_ measured by Differential Scanning Calorimetry (DSC) [8].

	Epoxy resin	Hardener	Phosphorus Content	T_g_	Char yield in N_2_
			(wt %)	(°C)	(%)
**DOPO**	DEN 438	DDM	0.81	-	-
**DOPO**	DGEBA	DDM	3.06	108	-
**DPPO**	DEN 438	DDM	0.81	-	-
**DOPO2-TDA**	DEN 438	DDM	1.02	189	29.8
**DPPO2-TDA**	DEN 438	DDM	1.45	185	38.1
**DOPO-NQ**	DGEBA	BACy	2.07	173	18.1
**DOPO-NQ**	DGEBA	TBFDCy	2.03	210	18.1
**DOPO-NQ**	DGEBA	BIDCy	2.14	160	23.1
**DOPO-NQ**	DGEBA	ODDCy	2.11	170	21.9
**DOPO-HQ**	DGEBA	BADcy	2.06	170	17.0
**DOPO-HQ**	DGEBA	TBFDCy	1.69	161	14.7
**DOPO-HQ**	DGEBA	BIDCy	1.53	146	18.1
**DOPO-HQ**	DGEBA	ODDCy	1.48	167	18.3
**DOPO-HQ**	CNE	PN	1.10	183	31.0
**DPODB**	CNE	PN	2.83	203	31.2
**DPPA**	DGEBA	DDM	3.20	131	-

## 5. Conclusions

Since the implementation of environmental directives banning or limiting the use of halogenated compounds, the development of flame retardants has focused on more environmentally friendly alternatives. Even though TBBPA still represents a large share of the flame retardant market for EE applications, it is progressively being replaced by non-halogenated alternatives. The largest volume share of flame retardants is occupied by inexpensive additives such as ATH. However, the relatively high loading necessary to impart flame retardancy often has negative repercussions on the physical properties of the finished product. Combinations of nitrogen-based flame retardants such as melamine polyphosphate and metal phosphinates also demonstrated flame retardancy for PWBs. Phosphorus compounds have received a growing attention from both industry and academic community. Unlike other halogen-free alternatives, phosphorus compounds have demonstrated flame retardancy with relatively low loadings, regardless of their mode of addition. Phosphorus flame retardants generally act in both gas and condensed phase. They can also be chemically tailored to favour one mechanism or the other. DOPO bridged compounds imparted flame retardancy to novolac epoxy resin cured with DICY/Fenuron with < 1.0% phosphorus content. UL 94-V0 rating can be reached when novolac epoxy resin is prereacted with DOPO (1.6%P). There have been promising results dealing with phosphorus flame retardants for EE applications that are environmentally friendlier. However, the chemical addition of DOPO to the epoxy resin significantly decreases the T_g_ of the cured resin which is problematic for lead-free soldering. Hence, new multifunctional reactive DOPO derivatives, phosphorus containing oligomers and phosphorus-based curing agents that would not have a detrimental effect on the T_g_ of the cured resin are subject to extensive research. Such reactive DOPO derivatives or phosphorus containing oligomers will also answer the problem of leaching that occurs with the use of additive flame retardant. In addition, efforts are currently invested towards developing new halogen-free flame retardant systems investigating both new phosphorus containing structures as well as synergism with other existing inexpensive inorganic additives.
